# In-silico study of the cardiac arrhythmogenic potential of biomaterial injection therapy

**DOI:** 10.1038/s41598-020-69900-4

**Published:** 2020-07-31

**Authors:** William A. Ramírez, Alessio Gizzi, Kevin L. Sack, Julius M. Guccione, Daniel E. Hurtado

**Affiliations:** 1 Department of Structural and Geotechnical Engineering, School of Engineering, Pontificia Universidad Católica de Chile, Santiago, Chile; 2 Nonlinear Physics and Mathematical Modeling Lab, Department of Engineering, Campus Bio-Medico University of Rome, Rome, Italy; 3 Department of Surgery, University of California at San Francisco, San Francisco, CA USA; 4 Division of Biomedical Engineering, Department of Human Biology, University of Cape Town, Cape Town, South Africa; 5 Institute for Biological and Medical Engineering, Schools of Engineering, Medicine and Biological Sciences, Pontificia Universidad Católica de Chile, Santiago, Chile; 6 Millennium Nucleus for Cardiovascular Magnetic Resonance, Santiago, Chile

**Keywords:** Cardiac device therapy, Computational science, Biomedical engineering

## Abstract

Biomaterial injection is a novel therapy to treat ischemic heart failure (HF) that has shown to reduce remodeling and restore cardiac function in recent preclinical studies. While the effect of biomaterial injection in reducing mechanical wall stress has been recently demonstrated, the influence of biomaterials on the electrical behavior of treated hearts has not been elucidated. In this work, we developed computational models of swine hearts to study the electrophysiological vulnerability associated with biomaterial injection therapy. The propagation of action potentials on realistic biventricular geometries was simulated by numerically solving the monodomain electrophysiology equations on anatomically-detailed models of normal, HF untreated, and HF treated hearts. Heart geometries were constructed from high-resolution magnetic resonance images (MRI) where the healthy, peri-infarcted, infarcted and gel regions were identified, and the orientation of cardiac fibers was informed from diffusion-tensor MRI. Regional restitution properties in each case were evaluated by constructing a probability density function of the action potential duration (APD) at different cycle lengths. A comparative analysis of the ventricular fibrillation (VF) dynamics for every heart was carried out by measuring the number of filaments formed after wave braking. Our results suggest that biomaterial injection therapy does not affect the regional dispersion of repolarization when comparing untreated and treated failing hearts. Further, we found that the treated failing heart is more prone to sustain VF than the normal heart, and is at least as susceptible to sustained VF as the untreated failing heart. Moreover, we show that the main features of VF dynamics in a treated failing heart are not affected by the level of electrical conductivity of the biogel injectates. This work represents a novel proof-of-concept study demonstrating the feasibility of computer simulations of the heart in understanding the arrhythmic behavior in novel therapies for HF.

## Introduction

Heart failure (HF) is a severe, chronic and progressive condition whose prevalence is expected to increase worldwide in the next decades. The total cost of HF in the United States alone is projected to be 69.7 billion dollars by 2030, representing approximately 244 dollars for every US adult. Even though survival after diagnosis of HF has improved over time with better treatments and dedicated devices, 50% of people die within five years after diagnosis^1^. For some patients with severe, progressive HF, transplant is the only viable option. Recent development of alternative treatments to the prevent of HF after myocardial infarction (MI) include the injection of biomaterials to restore cardiac function^2,3^. Pre-clinical trials suggested that injection of passive biopolymers can reduce remodeling in post-MI hearts and, at the same time, improve the left ventricular (LV) function. Algisyl-LVR is a commercial alginate hydrogel implant that has shown to improve function of the heart restoring a physiological LV anatomy^4^, while being feasible and safe in patients with critical HF at the time of surgery. Algisyl-LVR is injected into the mid-wall of the left ventricle, creating a solid rounded inclusion after consolidation^5^. Recent studies have shown that Algisyl-LVR injections stimulate the genesis of a fibrotic encapsulation that restrains the adjacent cardiomyocytes around the surface of the implanted biogel^6^. The constraint effect results in the prevention of left-ventricular dilation, which in turn reduces the cardiac wall stress^7^, which has shown to improve cardiac function and reduce maladative cardiac remodeling in swine models of MI^8^.

Patients with severe HF are at increased risk of ventricular tachycardia (VT), that can quickly evolve in ventricular fibrillation (VF), leading to sudden cardiac death. VT and VF are related to abnormal impulse propagation within the heart, namely spiral (in 2D) and scroll waves (in 3D)^9^. The primary source of such abnormalities is related to irregular spatiotemporal dispersion on the electrical properties at the cellular and tissue levels, such as the major ionic currents, action potential duration (APD) and action potential restitution^10,11^. The associated structural and electrophysiological changes occurring after MI act as a substrate for arrhythmia^12^. Because patients eligible for biomaterial injection therapy are already exposed to the development of cardiac arrhythmia, it is decisive that the treatment does not increase their susceptibility to the formation of an anomalous electrical propagation within the myocardium.

Recently, the mechanical response of infarcted hearts to biomaterial injections was quantified using predictive computational modeling^13^. Three-dimensional finite element analyses informed by echocardiography data from sheep hearts were carried out to compare different material properties of infarcted and healthy regions, showing that the presence of tissue filler significantly reduces myofiber stresses^14^. Idealized ellipsoidal LV models have also been used to measure the mechanical effects of different biomaterials, identifying the optimal distribution of injectates in terms of mechanical power^15^. High-resolution ex-vivo data was used to show that biopolymer injections act as an LV mid-wall constraint mechanism that prevents adverse remodeling in the heart without secondary effects on the cardiac function^7^. While these studies helped to elucidate the promising outcomes of biomaterial injections from a mechanical point of view, their role in cardiac electrical behavior remains poorly investigated. Few recent animal studies have done so. In one study, conductive biomaterials aimed at restoring impulse propagation in rat hearts reduced the QRS interval, suggesting improved electric conduction after MI^16^. Hydrogel injections, with a high degree of intra-myocardial spread, did not cause significant electrical abnormalities in rat hearts^17^. The knowledge acquired from experimental approaches is still limited because of the complex nature of these treatments and the strong constraints in measuring electrical propagation *in vivo*.

The vast literature about computational models in cardiac electrophysiology has made it possible to simulate most of the complex mechanisms leading to cardiac arrhythmogenesis, particularly when using highly-detailed anatomical models of the heart^18,19^. For instance, through MRI-based canine ventricular geometries, the arrangement and size of the peri-infarct (border) zone (BZ) were shown to be related to electrical excitation wavebreaks and onset of subsequent arrhythmias^20^. Detailed electrophysiological models of human ventricles were used to study the morphology of VF, confirming that VF dynamics mainly depend on APD restitution properties^21^. In-silico studies of histologically-based rabbit heart models with infarction were used to develop indices for measuring vulnerability to VT, which were previously validated in clinical applications and optical mapping^22^. Prediction of electrophysiological behavior of cell-based heart repair was addressed using 3D whole-heart modeling to explore the sustainability of VF of these treatments, demonstrating the promising outcomes of computational modeling for evaluating alternative therapies for HF^23^. More specifically, patient-specific in-silico studies have allowed the quantification of scroll-wave filaments arising during VF^24–27^, and their association to the effectiveness of defibrillation therapies^28–30^. This knowledge takes high relevance in the clinical management of failing hearts, as current clinical guidelines recommend implantable defibrillators as therapy for primary prevention of sudden cardiac death after MI^31,32^. The capabilities of using computational models to study the electrical behavior of infarcted hearts have been demonstrated, but have not been used to assess the potential role of biomaterial-injection treatments in the arrhythmic behavior of treated subjects.

In this work, we investigated the electrical behavior and arrhythmic potential of swine hearts treated with biomaterial injections by means of computational modeling. To this end, we used high-resolution DT-MRI images of swine hearts treated with Algisyl-LVR to create a computational model that represent the biventricular cardiac anatomy as well as the myocardial fiber orientations. We modeled and parameterized the transition zone from infarcted tissue towards the healthy tissue and modeled local tissue heterogeneities from MRI, accounting for injectate volumes. To account for transmural dispersion of repolarization, we divided the heart walls into three layers with endocardial, mid-myocardial, and epicardial cells, and modeled their distinctive behavior using a biophysical cellular model with specific properties for each layer. By performing an extensive computational campaign, we quantitatively characterized the electrical restitution properties of treated and untreated heart models and their performance during VF conditions. To do this, we developed a numerical method to compute important parameters such as activation time (AT) distributions, diastolic interval (DI) distributions and filament counting during simulations. Moreover, to assess the regional dispersion we constructed probability density functions of the APD restitution curve in different regions of the heart. Finally, we examined how the passive electrical properties of the injections influenced the long-term dynamics of VF for the treated heart models. By using computational modeling, this research assesses for the first time the potential of biomaterial injections to become a substrate for arrhythmia and their influence in the dynamics of VF.

## Results

**Figure 1:**
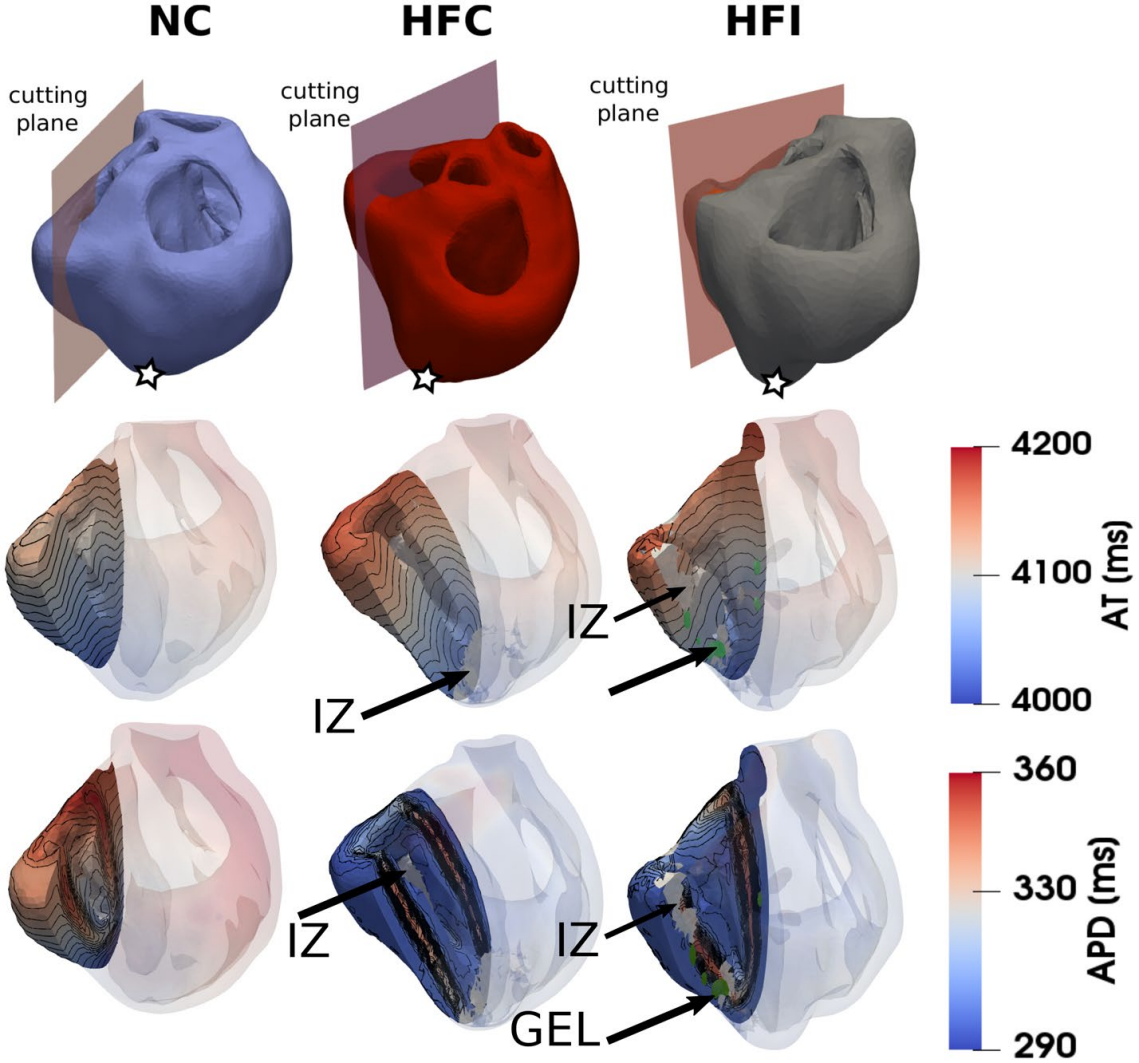
Spatial distribution of activation time (AT) and action-potential duration (APD) as measured after the fifth stimulus delivered at the apex. (Top row) computational model showing the biventricular geometry and cutting planes, with stimulation sites indicated with a star. (Middle row) AT maps for the selected cutting planes. (Bottom row) APD maps for the selected cutting planes. Infarcted zones (IZ) and bio-gel injections (GEL) are indicated with arrows.

A restitution protocol simulation was performed in three different computational heart models: a normal control heart (NC), a heart control with ischemic heart failure without biomaterial treatment (HFC), and a heart with ischemic HF and biomaterial injections (HFI). Figure 1 shows isochrone maps of the AT and APD, corresponding to the fifth stimulus delivered for all the three hearts. The spatial distributions of AT in the LV in all three cases look smooth and display a clear gradient along the apicobasal direction. The APD maps in the normal heart display a transmural heterogeneity associated to the three layers with different cell types included in the model, see Fig. 9. The HFC and HFI cases display a stronger transmural dispersion in APD, with lower APD values towards the epicardium and endocardium than the NC case.

**Figure 2:**
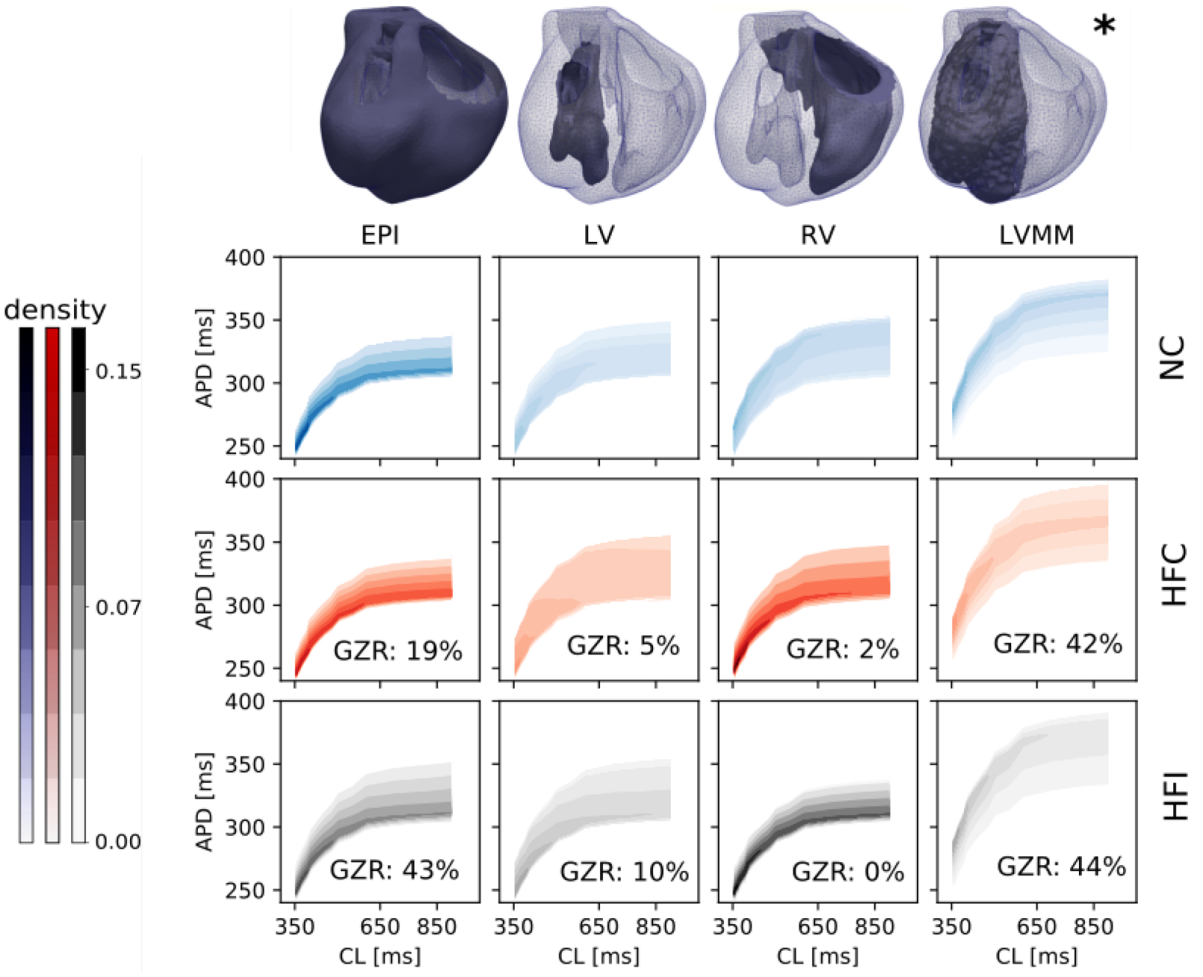
Restitution curve of the normal (NC), untreated (HFC) and treated (HFI) heart for the epicardial (EPI), left ventricle (LV), right ventricle (RV) and left ventricle mid-myocardium (LVMM) regions. Gray zone ratio (GZR)is reported for the HFI and HFC hearts. In general, higher dispersion of APD is found in the LVMM region.

**Figure 3:**
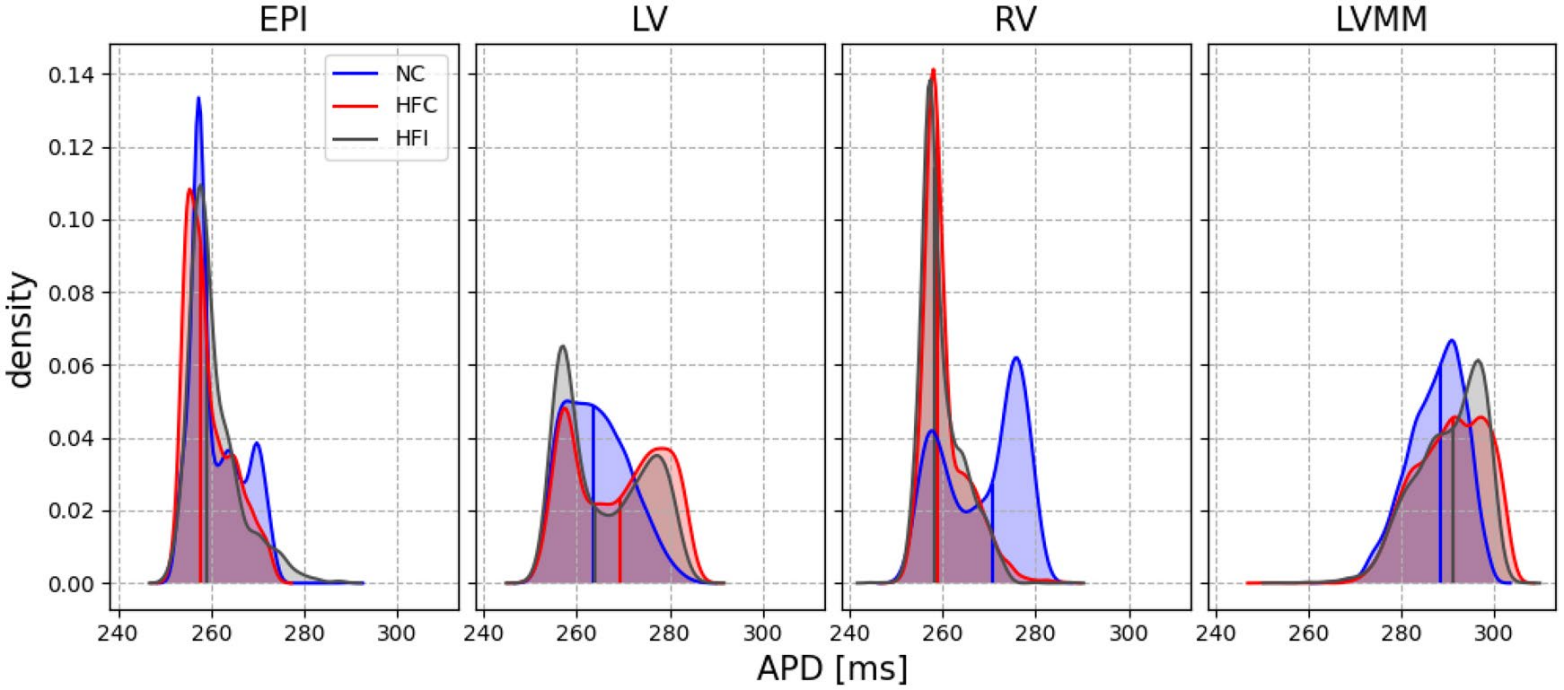
Empirical probability density functions of regional APD at CL$=370\,\text {ms}$. Median values are plotted with vertical lines. All three models result in similar distributions for the EPI and LVMM, while the NC case markedly differs from the HFC and HFI cases in the LV and RV regions.

Given the heterogeneous distribution of the APD within the myocardium, the relation between the APD and the cycle length (CL) was computed for the surface of the epicardium (EPI), the left-ventricle endocardium (LV), right-ventricle endocardium (RV) and left-ventricle mid-myocardium (LVMM). Figure 2 displays these regions, as well as the empirical probability density functions of the restitution curves on each of these surfaces. Each panel of Fig. 2 also shows the grey zone ratio (GZR), defined as the area of grey zone over the total area of the surface under consideration. The GZR was large ($> 40\%$) in the LVMM and EPI surfaces of the HFI heart, and in the LVMM surface of HFC heart. The GZR was low ($\le 10\%$) in all other surfaces, except for the EPI surface of the HFC heart. In all cases, restitution curves monotonically converged to APD = $250\,\mathrm{ms}$ as the CL approached a value of $350\,\mathrm{ms}$. In general, dispersion in APD increased as CL increased, with the largest dispersion found in the LVMM region in all three cases studied for high CL values. Figure 3 shows APD empirical probability density functions for the particular case of CL$=370\,\mathrm{ms}$. Qualitatively, all three heart models resulted in similar distributions of APD for the EPI and LVMM regions, while marked differences in the distribution shape were found for the NC case when compared to the HFC and HFI cases in the LV and RV regions. This trend is confirmed by comparing median values of the distributions, see Supplementary Table 1. A similar analysis on the distribution of APD for higher CL values confirms this trend, see Supplementary Fig. 1.

**Figure 4:**
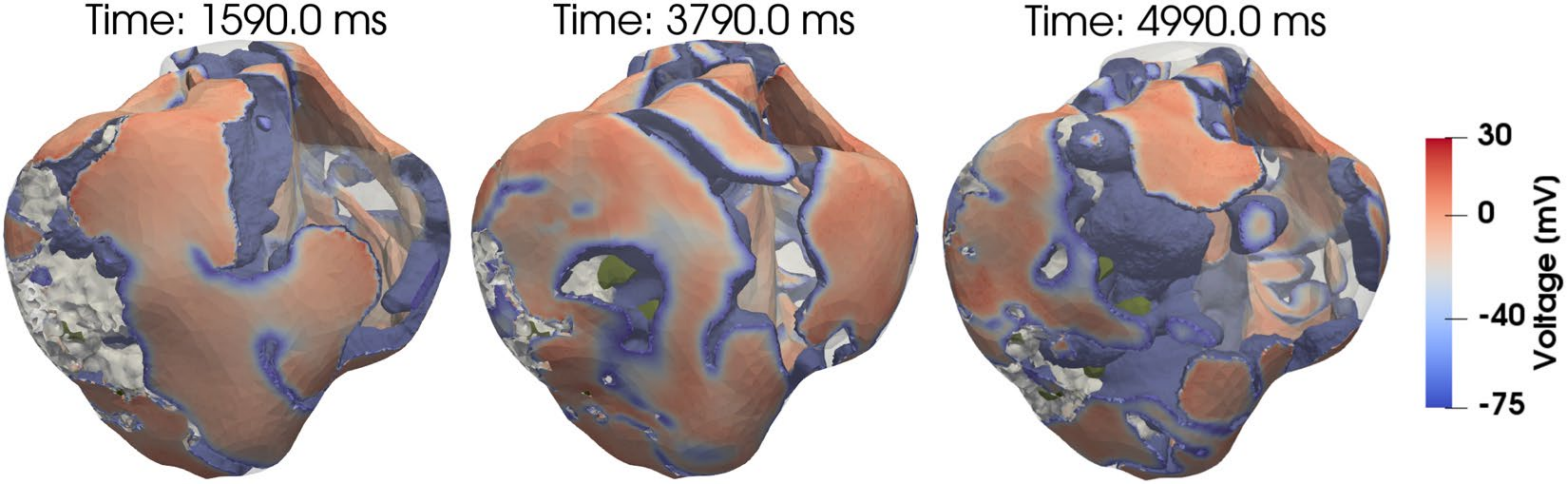
Temporal evolution of ventricular fibrillation in the HFI heart. Electrically-active regions ($V_m > -75.0\, \mathrm{mV}$) are depicted according to the color scalebar. IZ is depicted in grey, and biomaterial injections are depicted in dark green. The S1 stimulation site is the septum LV endocardium, while the S2 stimulus is delivered at the posterior zone of the epicardium. Rotors rapidly increase in time, and constantly interact with regions where biomaterial injections are located.

**Figure 5:**
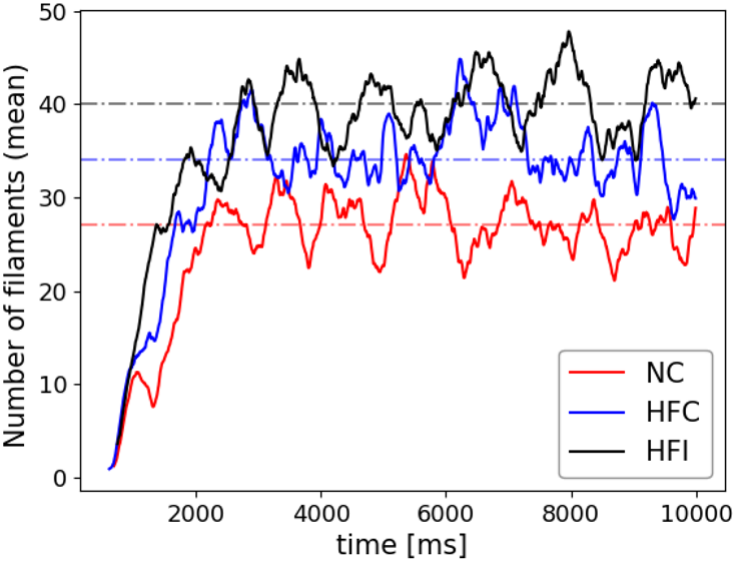
Evolution of the number of filaments for all three hearts (solid lines). Dashed lines correspond to the time-averaged number of filaments after the first 2500 ms. The time-averaged number of filaments in the NC, HFC and HFI subjects was 27, 34 and 40, respectively.

We studied VF sustainability in all hearts via an S1–S2 stimulation protocol for induction of VF. Figure 4 shows the depolarized (excited) tissue during the temporal development of arrhythmia in the HFI model, highlighting how the number of scroll waves rapidly increases as the time progresses. To induce VF, increasing levels of injected currents were employed on the different hearts until multiple scroll waves were achieved. In our study, the ratio of the NC, HFC and HFI S1–S2 stimuli amplitude was 15:11:11, and the vulnerable window was 408 ms, 406 ms and 423 ms for the NC, HFC and HFI subjects, respectively. To provide a quantitative indication of VF dynamics and sustainability, we assessed the time evolution of scroll waves by identifying the total number of 3D filaments at each time instant during a time window of 10 s, see Fig. 5. In all cases, the number of filaments stabilized after roughly 2500 ms. After that time, the NC, HFC and HFI subjects resulted in 27, 34 and 40 filaments, respectively.

**Figure 6:**
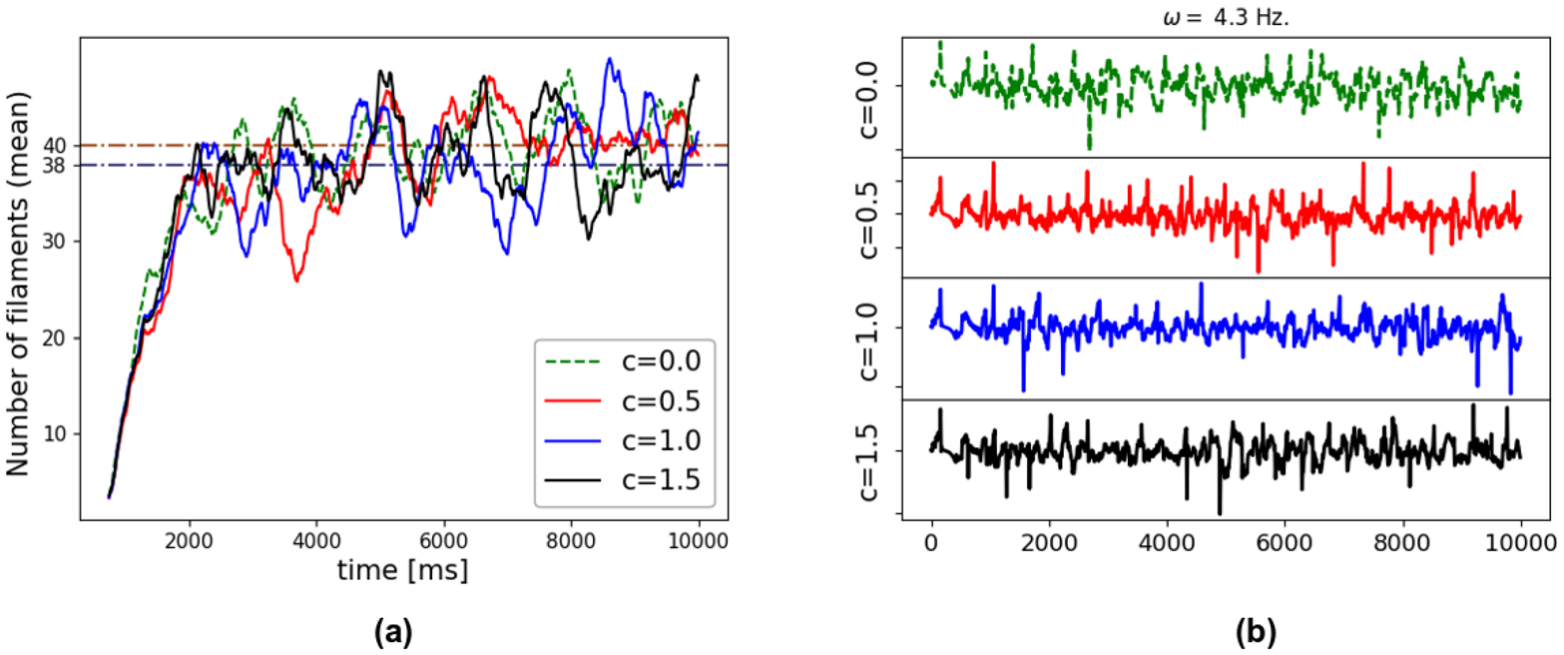
(**a**) Time evolution of the number of filaments during VF simulations of the HFI model for varying levels of electrical conductivity of the gel injections, parameterized by the value of the *c* ratio. The average value after 2500 ms is depicted with dashed lines. There are not substantial differences between each case: the average number of filaments after 2500 ms found in these simulations was 40, 40, 38 and 38 for the cases of gel conductivity ratios of 0.0, 0.5, 1.0 and 1.5, respectively. (**b**) Pseudo-ECG computed for each case.

To understand the potential impact of the electrical properties of gel injectates in VF sustainability, we performed a sensitivity analysis where the number of filaments during VF was studied for different levels of gel conductivity. We considered four levels of gel conductivity by setting $c = \{0.0, 0.5, 1.0, 1.5\}$, where *c* is defined as the ratio between the gel conductivity and the normal tissue conductivity. Figure 6a shows the time evolution of the number of filaments for the HFI model where stabilization is achieved after 2500 ms, resembling the convergent behavior observed in Fig. 5. The average number of filaments after 2500 ms found in these simulations was 40 for the cases of $c = \{0.0, 0.5, 1.0 \}$ and 39 for the case of $c=1.5$. Pseudo-ECGs for all cases are reported in Fig. 6b, where the fundamental frequency was $4.3\,\mathrm{Hz}$ in all cases, regardless of the biogel conductivity assumed. The volume of the injected biomaterial represented roughly $3\%$ of the heart total conductive volume.

## Discussion

In this work, we present a novel *in silico* pilot study of the influence of biomaterial injection therapy on the arrhythmic behavior of failing hearts. We successfully developed a robust and computationally-efficient method that allows high-resolution subject-specific MRI and DT-MRI data to be used to evaluate the regional distribution of the APD restitution curve and the number of filaments formed during VF of control and treated hearts. We note that our study represents a proof-of-concept study with only one subject per group, and therefore is not sufficiently powered to make inter-group conclusions. Despite this limitation, it represents a unique effort to understand how the dispersion of repolarization and VF dynamics behavior change in failing hearts treated with alginate hydrogel injections, as no electrophysiological studies for this treatment have been reported to date.

Simulations of standard restitution protocols suggest that there are important differences in the spatial distribution of AT and APD between the normal heart and both hearts with ischemic HF, treated and untreated. Spatial heterogeneity in the APD was observed in all three hearts, with a marked transmural gradient that can be explained by the use of different ionic cellular models for the endocardium, mid-myocardium and epicardium regions in the heart, see Figs. 1 and 9. The regional heterogeneity in APD can be also confirmed from the empirical probability density functions sampled from the restitution curves in selected regions in the heart, see Fig. 2. In all cases, the largest APD mean values and dispersion were found at the LVMM region, which can be partly explained by the larger APD displayed by mid-myocardial cells, see Fig. 9. Despite the fact that the LVMM presented the highest ratios of gray zones in HF hearts, the APD probability distributions did not seem to be affected when comparing normal and failing hearts, a trend that is confirmed when analyzing the APD distributions for different values of CL during restitution protocols in this same region, see Fig. 1 in the Supplementary Materials. In contrast, the LV and RV regions in the HFC and HFI subjects markedly differed from the NC subject. In the LV region, failing hearts displayed a bimodal distribution with higher dispersion (standard deviation) and median APD than the normal heart, which displayed a unimodal distribution. An inverse trend was found in the RV region, where the normal heart resulted in a bimodal distribution with higher median and dispersion than both the untreated and treated failing hearts, which displayed unimodal distributions, see Tables 1 and 2 in the Supplementary Materials. These findings suggest that, while the development of HF does result in marked changes in the dispersion of repolarization, the use of biogel injection treatment does not seem to affect the spatial dispersion of APD in failing hearts. Further, we attribute the differences in dispersion of repolarization in failing hearts when compared to the normal case to the remodelling that typically takes place after MI. This conclusion is supported by previous work using 2D simulations of cardiac tissue under an S1–S2 protocol, where the dispersion of APD was correlated with the formation of abnormalities in the electrical activity, such as VT. In these simulations, regions with altered restitution properties co-localized with zones with wave break and reentry^33,34^. Figure 2 shows that the restitution curve distribution at small CL values preserves a similar behavior for all hearts. This is an important observation since an anomalous behavior of the restitution curve at lower CL values is related to the formation of action-potential alternants and consequent wavebreaks^35–37^. For instance, experimental studies in human hearts with ischemic HF in the LV have shown that altered dynamics of the restitution curve at high pacing frequencies lead to electrical instabilities^38^.

We have studied the VF dynamics in normal, untreated HF and treated HF subjects. In all three cases, we reached conditions for inducing and sustaining fibrillation by varying the location and duration of the excitation. We note that the injected current needed to induce VF in the NC case was considerably higher than in the HFC and HFI cases, which is consistent with experimental and clinical observations. During the sustained fibrillation regime, the average number of filaments in the HFC and HFI hearts stabilized around 34 and 40, respectively (Fig. 5). The NC heart reaches a markedly lower value of 27 filaments during sustained VF. These results show that untreated and treated failing hearts resulted in increased VF sustainability when compared to a normal heart. Further, the larger number of filaments in the HFI subject compared to the HFC subject suggests that the biogel treatment can result in higher VF sustainability than that expected for untreated failing hearts. This observation has important implications in the development of biomaterial-based treatments, especially when considering defibrillation procedures. Computational investigations have shown that a successful defibrillation process requires less energy when fewer filaments are present within the tissue^39^. Since the HFI resulted in a larger number of filaments than the HFC heart, higher defibrillation-energy levels may be required in treated hearts^29^. Since ischemic HF biomaterial-treated hearts are already at high risk of developing arrhythmias, these results provide crucial knowledge to be considered in the experimental design of defibrillation treatment of hearts with gel injectates.

A mechanistic explanation of the higher sustainability of VF observed in treated and untreated hearts can be supported by the changes in dispersion of repolarization resulting from remodeling. Post-MI HF results in marked alterations in key structural features of the heart such as cardiomyocyte principal orientation as well as changes in the cardiac volume^6^. These features induce a higher dispersion of repolarization, materialized in our case in critical changes in the distribution of APD in the RV and LV regions. Bimodality of the LV provides a wider substrate for discordant alternans and wavebreaks because of the larger volume of conductive mass available in the LV that can accommodate a large number of spiral waves^40^. We remark that such an information is not directly inferrable from classical restitution curves, usually quantified only at specific locations, but it is crucial to connect the analysis of APD restitution distributions with an intrinsic spatial feature (electrotonic and memory effects) given by the spiral core filament in our analysis^11,37,41^.

In the first set of simulations, we treated biomaterial injections as non-conductive regions within the myocardium, which may not be the case for a biocompatible material. To assess the validity of this assumption, we developed a sensitivity analysis study where injections within the myocardium were varied to understand the effect of gel conductivity in our results. We found that gel conductivity does not alter VF dynamics, as shown in Fig. 6a. This result is further supported by the pseudo-ECG simulation reported in Fig. 6b, where we found a fundamental frequency that does not depend on the level of gel conductivity. Further investigations concerning the conductivity properties of biomaterial injections are needed in order to fully characterize their significance in different electrophysiological models and multiple clinical conditions. For instance, biohybrid hydrogels composed of collagen, alginate and poly:polystyrene sulfonate have shown to be electroconductive, preventing arrhythmia in cardiac tissue constructs from neonatal rat hearts^42^.

This work has limitations that should be addressed in future extensions. An important limitation is that only one subject per group was considered, which is largely justified by the high computational burden, both in wall-clock time and in required infrastructure, that each simulation demands. While each group necessitates a larger population in order for the results to be statistically meaningful, we remark that the aim of this work is to present a proof-of-concept study that demonstrates the feasibility of performing pre-clinical studies of biomaterial injection in-silico. In particular, our results can be used as preliminary data in the design of future computational studies. In such an effort, the electrophysiological cellular model will require dedicated measurement and fine-tuning of the restitution parameters to set the spatiotemporal dynamics within a patient-specific framework. Further, it is worth noting that the behavior of interfaces between healthy myocardial tissue and gel biomaterials necessarily imply alterations in the local reaction dynamics and conduction properties, which currently remains an open topic from the experimental and theoretical perspectives^8^. These alterations may resemble the situation of border zones located near the boundary of the IZ, where strong cardiac remodelling is observed. These border zones reportedly play a meaningful role in the propagation of action potentials, since they may promote the formation of abnormalities such as action potential alternans^10^ and arrhythmias^43^. While recent multiscale models of cardiac tissue have been able to theoretically link the remodelling of gap-junction conductivity with reduced conduction velocity in cardiac tissue^44^, further studies should quantify in biophysical terms the level of remodelling found at the gel-intact tissue interface, in order to incorporate additional nonlinearities in the emerging cardiac behavior^45–48^. Another limitation is the absence of electromechanical coupling in our simulations. Because Algisyl-LVR injection treatments are specifically developed to deliver passive mechanical support to the ventricle, the electromechanical coupling plays a decisive role to examine the overall performance of treated hearts. Computational models that incorporate electromechanical coupling have become increasingly relevant given the role of deformation in the local electrical behavior and spatial propagation of electrical impulses, particularly in VF dynamics^49–53^. Therefore, future efforts should include electromechanical coupling in order to better characterize the behaviour of biomaterial-treated hearts, at the expense of increasing the computational costs.

Future work may help to elucidate how biomaterial injection treatments can be enhanced such that the overall function of HF hearts could be improved without affecting their electrical performance. For instance, future efforts could focus on understanding how the distribution of injections can affect the electrophysiological behavior of treated hearts. Recent studies show that the volume and location of biomaterial injections correlate with the reduction of pathological conditions within the heart^54^. Another avenue of research is to extend the current simulations to include electrophysiological models that could take into account the multiscale nature of the myocardial tissue, and muscle contraction.

## Methods

### Geometrical and morphological representation of swine hearts

This study considered one normal control heart (NC), one heart control with ischemic heart failure without biomaterial treatment (HFC), and one heart with ischemic HF and biomaterial injections (HFI). These subjects were selected as representative of larger cohorts considered in a previous morphological study of the effects of fiber remodeling under biogel treatment^6^. The three selected subjects included natural geometrical differences due to biological variance and remodeling to ischemia. This has been quantified in previous studies which showed local wall thinning and fiber reorientation due to ischemia, but that the global structure and morphology of the hearts were not significantly different^6^. The injection protocol for the hydrogel delivers a total of 12–14 intra-myocardial injections (0.3 mL each) in a circumferential pattern into the LV free wall during an open chest procedure. These are administered in roughly 1.5 cm apart and in two rows: one above and one below the mid-ventricular plane between the base and the apex. The hydrogel, which accounts for roughly 3% of the total LV wall volume, does not disrupt fiber orientation, but rather conforms to the native structure it is injected into, forming ellipsoidal shapes orientated with the local fiber structure^6^. The solidified injections mitigate the effects of adverse remodeling by anchoring and supporting the surrounding tissue in the nearby vicinity of the circumferential pattern. A deeper analysis of therapy efficacy and the mechanism of action in mechanical terms was recently provided in a recent study^7^.

**Figure 7:**
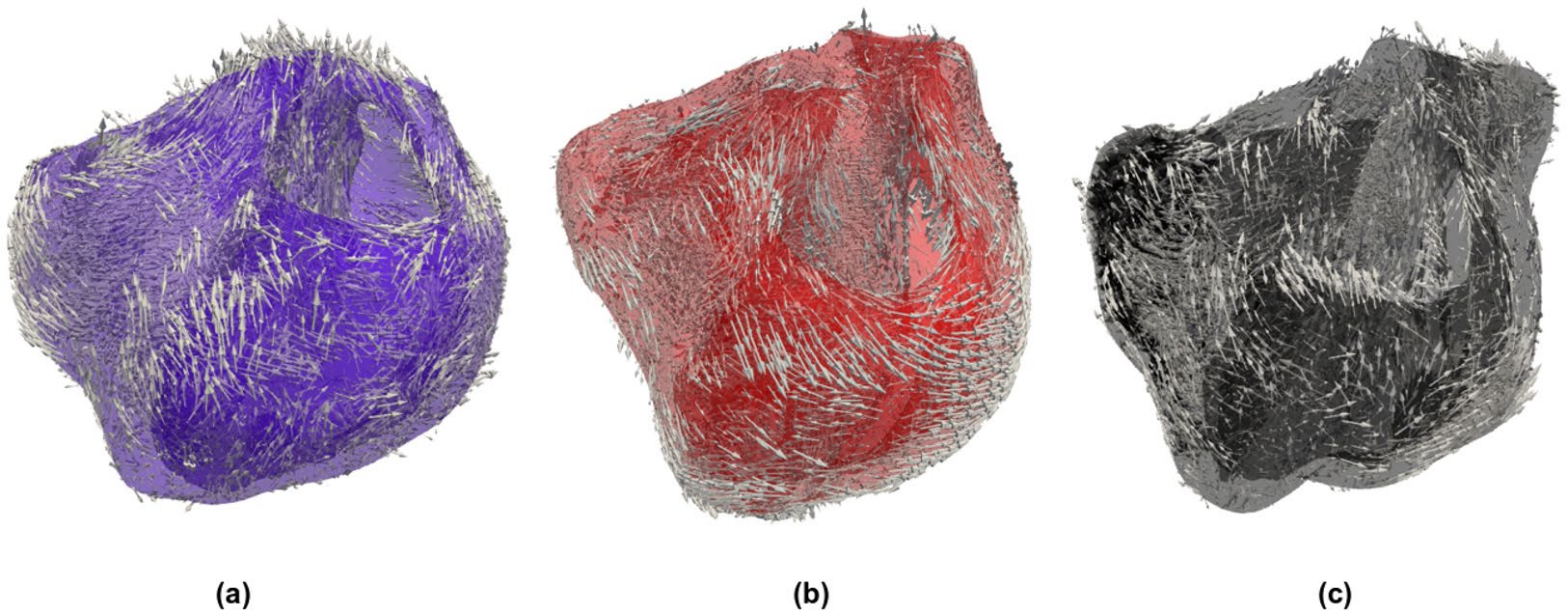
Heart geometry and fiber directions: (**a**) normal control (NC) heart, (**b**) heart-failure heart (HFC), (**c**) heart-failure heart treated with biomaterial injections (HFI). The geometry was reconstructed from high-resolution magnetic-resonance images (MRI), and the fiber directions were obtained from diffusion-tensor MRI.

**Figure 8:**
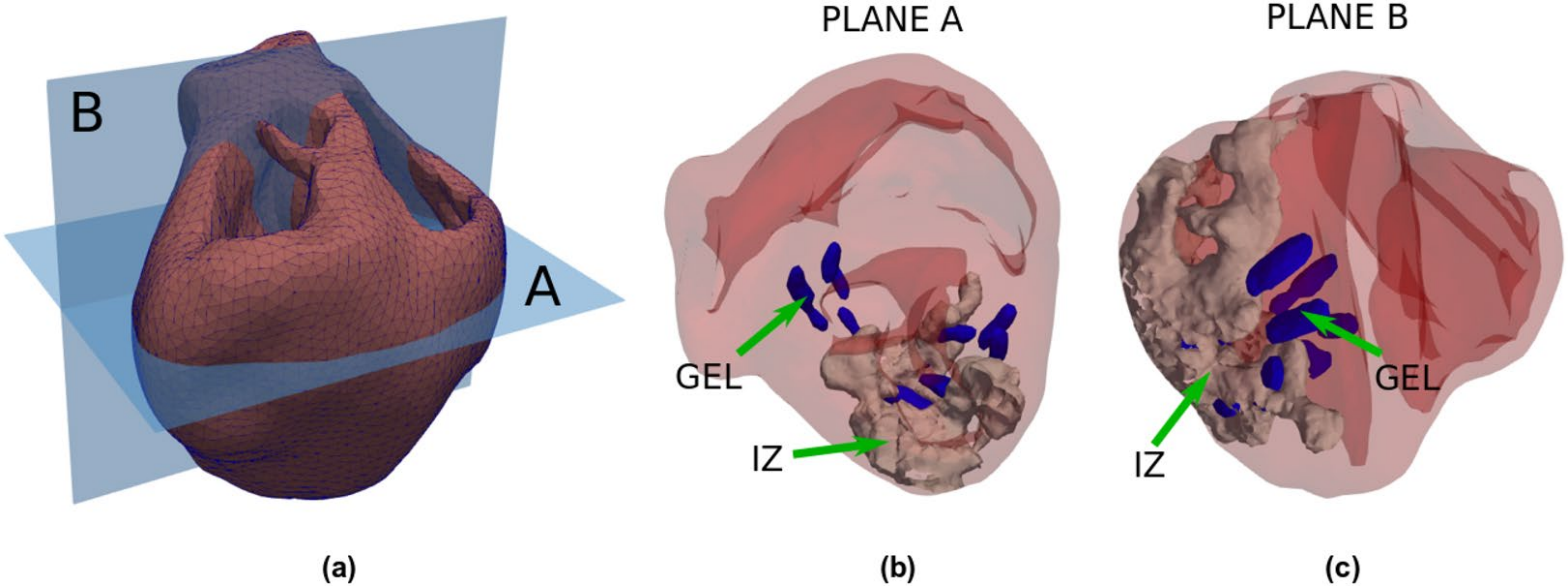
Detailed views of the heart-failure treated heart (HFI) in which biomaterial injection distribution is depicted in blue and infarcted regions are depicted in gray. Biomaterial injections are dispersed within the mid-myocardial left ventricle. Some injections are placed within the NZ region, and others are placed within the intersection of IZ and NZ regions.

**Figure 9:**
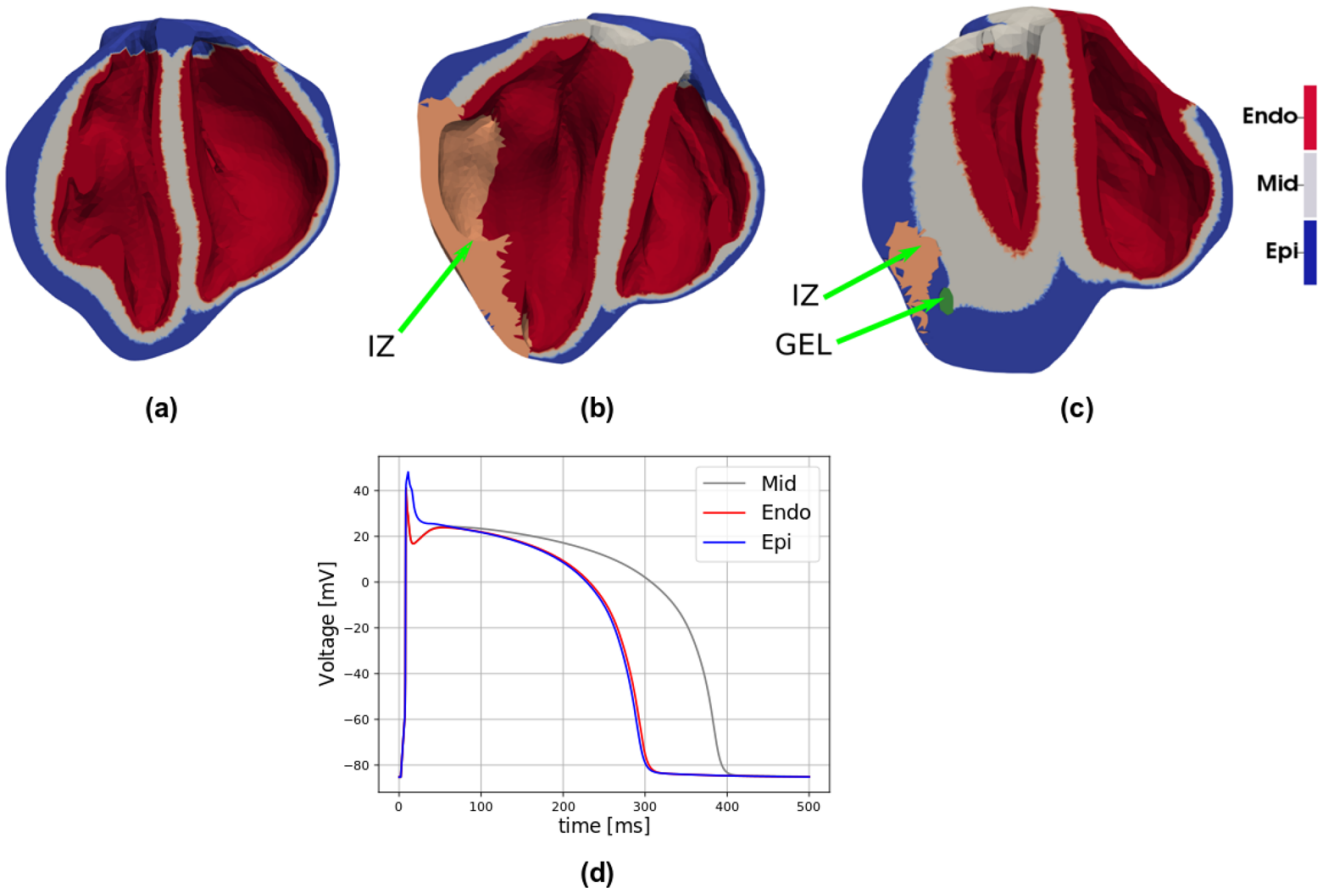
Hearts ventricles sections showing the endocardium (Endo), mid-myocardium (Mid), and epicardium (Epi) regions in (**a**) the NC model, (**b**) the HFC model, and (**c**) the HFI model. These domains are defined using a regional segmentation technique based on Laplace interpolations^56^. Each domain considers a specific cellular model for ionic transmembrane current. (**d**) Action potentials for the three type of cells considered in the construction of the heart models

Subject-specific accurate geometric representations of heart bi-ventricular structure, infarcted tissue, and biomaterial injections are used as the computational domain for the numerical simulations (Figs. 7 and 8). Imaging data originates from *ex vivo* segmentation of high-resolution MRI and DT-MRI of swine hearts. The experimental protocol, image acquisition, segmentation process and reconstruction methodology have been described previously^8,55^. In brief, myocardial infarction was induced by occluding the obtuse marginal branches of the left circumflex artery. Eight weeks after MI, animals underwent Algisyl-LVR injection, and hearts were excised eight weeks later. Anatomical MRI and DT-MRI were acquired using a readout-segmented diffusion-weighted spin-echo sequence with $1.0 \times 1.0 \times 1.0 \,\hbox { mm}^{3}$ spatial resolution. We discretized the heart domains using tetrahedral finite elements, identifying healthy, infarcted and hydrogel regions based on MRI observations (Fig. 7). Following previous works^55^, local properties for the infarcted and healthy tissues are modulated by the volume fraction of healthy tissue. This volume fraction is represented by the space-dependent scalar function $h=h(\mathbf{x}) \in [0,1]$, where $\mathbf{x}$ represents the Cartesian coordinate vector. In particular, $h(\mathbf{x})=0$ defines properties of the infarcted zone (IZ), $h(\mathbf{x})=1$ identifies healthy tissue, and $0<h(\mathbf{x})<1$ defines the transition zone or gray zone (GZ), where mixed electrical properties of infarcted and healthy tissue are modeled according to the literature^20,43^. A gray zone ratio (GZR) could also be computed from this function to characterize the differences in GZ distribution between each heart under study. Myocardial fiber orientation, based on DT-MRI, was assigned to the mesh nodes and interpolated inside each finite element, delivering a continuous spatial vector field representation of the cardiac fiber orientation $\mathbf {f}=\mathbf {f}(\mathbf {x})$ for each heart analyzed^55^. To account for transmural dispersion of repolarization, we used a Laplace’s interpolation method^56^ to divide the heart wall into epicardial, mid-myocardial, and endocardial layers using a thickness ratio of 2:3:3, respectively. Figure 9 shows these transmural layers for the three hearts analyzed. The LVMM surface used in the study of APD distribution was constructed from the Laplace’s interpolation by creating a mesh at the mid-surface of the mid-myocardial region.

### Numerical modeling of cardiac electrical activity

Given the cardiac domain $\Omega \in {\mathbb {R}}^3$ composed by conductive tissue, the propagation of electrical impulses within a time interval [0, *T*] was modeled using the monodomain electrophysiology equation derived from current balance^57^, which takes the form

1a $$\begin{aligned} \dfrac{\partial V_m}{\partial t}&= \nabla \cdot \left( \mathbf{D} \, \nabla V_m \right) + I_s(V_m,\mathbf{r}) \quad \text {in} \quad {\Omega \times [0,T]} \,, \end{aligned}$$

1b $$\begin{aligned} \dfrac{d\mathbf{r}}{d t}&= \mathbf{g}(V_m,\mathbf{r})\,, \end{aligned}$$

where $V_m:\Omega \times [0,T] \longrightarrow {\mathbb {R}}$ represents the transmembrane potential, defined as the difference between intracellular and extracellular voltages, $I_s$ is the sum of the ionic currents depending on the transmembrane potential and $\mathbf{r}:\Omega \times [0,T] \longrightarrow {\mathbb {R}}^{m}$ is the set of state variables. This monodomain framework is constructed from a standard bidomain model^58^ by assuming that both intracellular and extracellular spaces have equal anisotropy ratios. In an averaged macroscopic sense, the conduction velocity is faster along the fiber direction and slower in a direction transverse to it. In particular, the conductivity tensor ${\mathbf{D}} \in {\mathbb {R}}^{N \times N}_{+}$ is assumed to be transversely isotropic, which can be expressed as

2 $$\begin{aligned} \mathbf{D} = h(\mathbf{x}) \left[ d_{\perp } \mathbf{I} + \left( d_{\perp }-d_{\parallel }\right) \mathbf{f} \otimes \mathbf{f}\right] \,, \end{aligned}$$

where $d_{\perp },d_{\parallel }$ represent the transversal and longitudinal conductivities, respectively, and $\mathbf{f}=\mathbf{f}(\mathbf{x})$ is the fiber direction vector field. The conductivity ratio $d_{\parallel }/d_{\perp }$ varies typically between 4 and 9^59^. We selected a conductivity ratio of 4, establishing the longitudinal conductivity as $d_{\parallel }=0.1\;\mathrm{mm}^2/\mathrm{ms}$, with a corresponding transversal conductivity of $d_{\perp }=0.025\;\mathrm{mm}^2/\mathrm{ms}$. To complement equations (1a) and (1b), we assume the no-flux boundary condition by denoting the boundary normal by $\mathbf{n}$ and enforcing that

3 $$\begin{aligned} \nabla V_m \cdot \mathbf{n} = 0 \quad \text {on} \quad \partial \Omega , \end{aligned}$$

which includes the internal interfaces between cardiac tissue and gel injection, as the gel is initially assumed non-conductive. For the simulations where the biogel is assumed to be electrically conductive, we consider $\Omega _{gel}$ as the domain of injectates, and represent the electrical conduction in that region by the Laplace equation

4 $$\begin{aligned} \nabla \cdot \left\{ \mathbf{D}_{gel}\nabla V_m \right\} = 0 \quad \text {in} \quad \Omega _{gel}. \end{aligned}$$

In writing Eq. (4) we have considered $-V_m$ as the voltage field in the biogel. This consideration can be justified by assuming that the biogel domain constitutes an extracellular domain, and that the voltage in the intracellular domain is zero in $\Omega _{gel}$, allowing the use of the transmembrane potential field in the simulation of electrical conduction in the biogel domain.

The transmembrane ionic current is modeled according to the model proposed by ten Tusscher and Panfilov (TP06)^11^ for ventricular human cells. This model is fitted to reproduce APD restitution curves measured in humans, and have an extensive description of the intracellular calcium dynamics. It has been broadly used for the study of reentrant arrhythmia and electrical instability at cellular and tissue levels. In this model the ionic current density, defined as the sum of all transmembrane current densities, is given by

5 $$\begin{aligned} I_s = I_{Na}+I_{K1}+I_{to}+I_{Kr}+I_{Ks}+I_{CaL}+I_{NaCa}+I_{NaK} +I_{pCa}+I_{pK}+I_{bCa}+I_{bNa}, \end{aligned}$$

where $I_{NaCa}$ is $Na^+/Ca^{2+}$ exchanger current, $I_{NaK}$ is $Na^+/K^+$ pump current, $I_{pCa}$ and $I_{pK}$ are plateau $Ca^{2+}$ and $K^+$ currents, and $I_{bCa}$ and $I_{nNa}$ are background $Ca^{2+}$ and $Na^+$ currents. One advantage of using this model is that, by changing the parameter values it allows for the representation of transmural heterogeneity observed in myocardial tissue. To this end, the cardiac wall was divided into epicardial, mid-myocardial and endocardial layers using the distribution ratio 2:3:3 as adopted in previous works^60^, see Fig. 9. The set of parameters corresponding to each layer, which were used in this work for the simulation of the restitution protocol, have been reported elsewhere^11^. For the simulation of VF, the parameter values reported in^21^ were employed, which are included in Table 1. Initial values for the gating and internal variables are included in Table 2.

**Table 1:** Parameter values used in VF simulations with the TP06 model.

Section	$G_{Kr}$	$G_{Ks}$	$G_{pCa}$	$G_{pK}$	$\tau _f$ Inactivation
Midmyocardium	0.172	0.0515	1.8545	0.00073	$\times 2$
Epicardium	0.172	0.2205	1.8545	0.00073	$\times 2$
Endocardium	0.172	0.2205	1.8545	0.00073	$\times 2$

Parameters not included in this table take the same values reported in^11^.

Parameters that where modified were the maximum conductance of the $I_{Kr}$,$I_{Ks}$, $I_{pCa}$ and $I_{pK}$ currents. The time constant of the *f* gate was also modified.

**Table 2:** Initial conditions used in the TP06 model.

*V*	$Xr_1$	$Xr_2$	*Xs*	*m*	*h*	*j*	*d*			
$-85.23$	0.00621	0.4712	0.0095	0.00172	0.7444	0.7045	$3.373\times 10^{5}$			

*f*	$f_2$	*fCass*	*s*	*r*	$Ca_i$	${\bar{R}}$	$Ca_{SR}$	$Ca_{ss}$	$Na_i$	$K_i$
0.7888	0.9755	0.9953	0.999998	$2.42\times 10^{-8}$	0.000126	0.9073	3.64	0.00036	8.604	136.89

**Figure 10:**
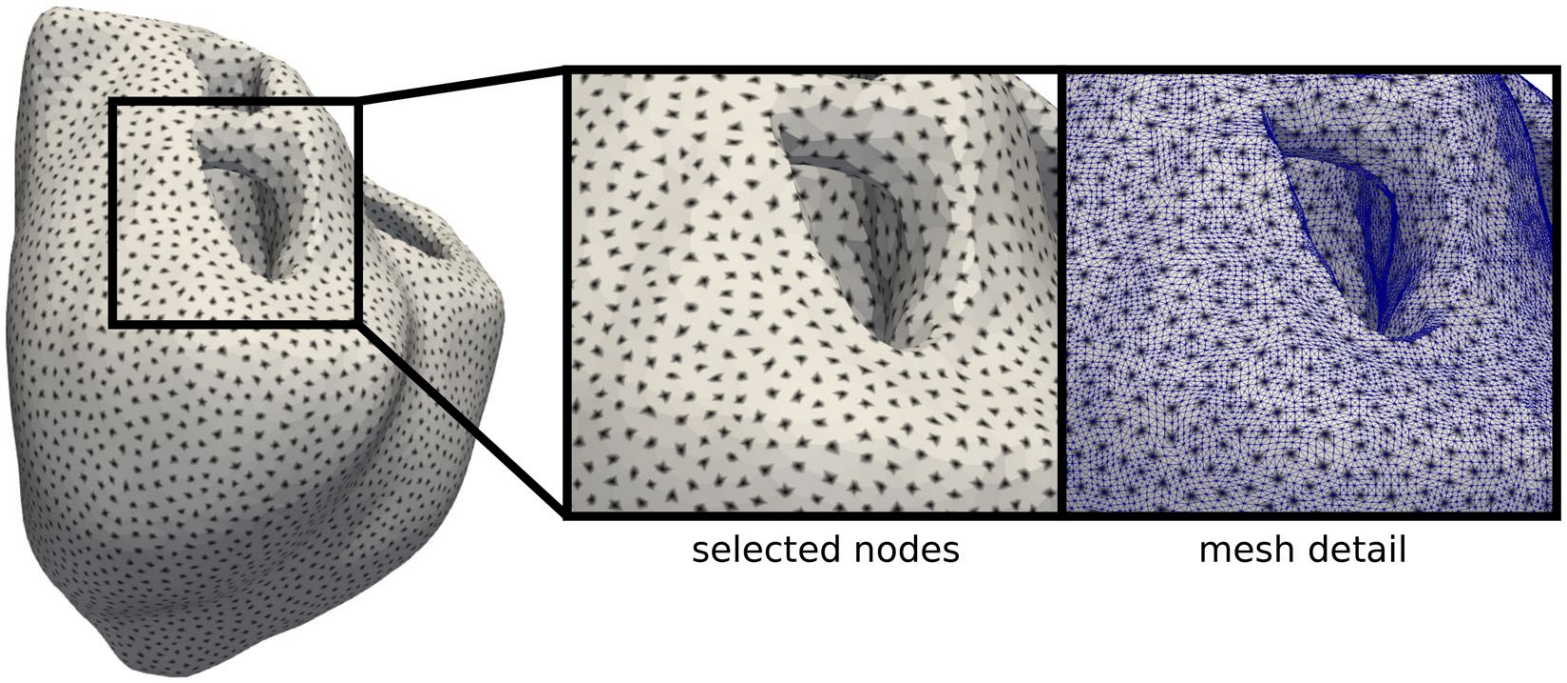
Detail of the nodes selected to compute the APD restitution curve (black dots) during simulation time, thereby reducing computational costs during post-processing. Regional distribution of the APD restitution is computed using the information taken from this set of nodes, which are selected based on unrefined meshes.

The governing equations described in Eq. (1a) are discretized in space using a standard Galerkin finite-element scheme^61^, where linear tetrahedral elements are employed to approximate the transmembrane potential field. Time integration was performed using an operator splitting method^58^. All numerical implementations were developed in Python, using the FENICS Project software and the *Cbcbeat* software collection in an in-house parallel computing platform. Given the well-known dependency of the conduction velocity to the mesh size, conduction velocity convergence was carried out for linear tetrahedral elements (Fig. 10). In particular, we found that a characteristic mesh size of $\approx 0.8\;\mathrm{mm}$ provides a good approximation of the sought conduction velocity ($\approx 0.67\,\mathrm{mm}/\mathrm{ms}$) at a normal pacing. This implies a set of nonlinear equations with over 7 million degrees of freedom. Time integration of gating evolution equations at quadrature points is performed using the explicit Rush-Larsen method with a time step of $dt=0.1\;\mathrm{ms}$. Overall, 1 second of simulation required about 4.8 hours of computation using 62 CPU’s within a parallel computing platform AMD Opteron Processor 6378. For the APD restitution estimation, this meant a total of 81.6 hours for each heart, whereas VF simulations involved around 48.0 hours of computation for each heart.

We developed in-house routines to post-process the simulation results to compute parameters such as activation (AT) and repolarization times (RT), during numerical simulations with a time step $dt^*=5dt$ over selected nodes in the finite element mesh (Fig. 10). The nodes were selected using information of unrefined meshes. This setup reduced computational costs, especially for post-processing purposes such as the APD restitution curve. Given the complex geometry of each model, we also performed region labeling for the different heart surfaces by using a Laplace interpolation approach. In particular, we computed APD restitution curves in the LV endocardium (LV), RV endocardium (RV), left ventricle mid-myocardium (LVMM), and epicardium (EPI) on a simple subdivision of the heart geometric features directly during simulations. Accordingly, we produced a probability density function (PDF) using a Gaussian kernel density approach to analyze the distribution of the APD restitution in these regions.

### Simulation protocols and analysis

To compute the APD restitution distribution, the three heart models were paced at the apex location (see star in Fig. 1) 32 times with varying pacing frequency. The total simulation time reached 17 s of physical time for each geometry model, with stimulus value of $40\, \mathrm{mV}/\mathrm{ms}$ and duration of $2\,$ms. At each pacing cycle length (CL), the APD was computed locally to obtain a regional distribution of the restitution curves. In particular, APD was computed for each selected node (see Fig. 10) of the finite-element discretization and the PDF was estimated from this data (see Fig. 2). For comparison purposes, the PDF was examined within the EPI, LV, RV and LVMM surfaces.

An S1–S2 protocol was implemented to induce VF in each heart model. The S1 stimulation site was the septum LV endocardium, while the S2 stimulus was delivered at the posterior zone of the epicardium, near the tail of the S1 wave. The timing between S1 and S2 was fine-tuned to obtain excitation wave-break, formation of a scroll-wave and evolution into VF. A vulnerable window was measured as the elapsed time between the S1 and S2 pulses for which a sustained VF was achieved ($> 2$ s). Depending on the heart model used, different values for the S2 stimulus were needed. The S2 stimulus value was $300\, \mathrm{mV}/\mathrm{ms}$ (7.5 times the S1 stimulus value) for the NC heart, while for both HFC and HFI hearts the S2 stimulus value was around $220\, \mathrm{mV}/\mathrm{ms}$ (5.5 times the S1 stimulus value). The vulnerable window was 408 ms, 406 ms and 423 ms for the NC, HFC and HFI heart, respectively. The complexity of activation patterns developed during VF dynamics was quantified by computing the number of rotors in time. These rotors are 3D scroll waves that rotate around a filament^24,62^. Scroll wave filaments were distinguished using the algorithm proposed by Fenton and Karma^59^. In particular, a singular point is found by computing the intersection of an isopotential line (-70 mV) with the condition $dV_m/dt =0$. In the computational model here implemented, each singular point is related to a single finite element. Afterwards, scroll wave singular line, e.g. filament, was identified and labeled by using a density-based spatial clustering algorithm (DBSCAN)^63^. This method allows one to group elements that are closely packed together forming a specific filament. Each group (filament) formed is classified, updated and counted every $10\,$ms of physical time directly during simulations.

The electrical properties of alginate hydrogel implants have not been reported to date, thus in the previous experiments we have assumed that its conductivity is zero. To assess the effect of gel conductivity on VF sustainability in the treated heart, we performed a sensitivity analysis where the biogel is assumed to behave as a passive conductor. VF simulations were performed assuming an isotropic conductivity in the gel of the form

6 $$\begin{aligned} \mathbf{D}_{gel} = c d_{\perp } \quad \text {in} \quad \Omega _{gel}, \end{aligned}$$

where *c* is a conductivity ratio that modulates the conductivity of the gel based on the value used for transversely-isotropic propagation in normal cardiac tissue. Accordingly, for values of the conductivity ratio $c<1$ the biomaterial region is less conductive than the normal tissue and for values of conductivity ratio $c>1$ the biomaterial region is more conductive than the normal tissue. We performed simulations for $c = \{0.0, 0.5, 1.0, 1.5\}$, and computed the number of rotors in each case to evaluate the spatiotemporal dynamics during VF. pseudo-electrocardiograms (EGCs) were computed by estimating the surface potential using the approximation^64^

7 $$\begin{aligned} V_e= - \int _{\Omega } \nabla V_m \cdot \nabla \frac{1}{||\rho ||} d\Omega ,\quad \text {with} \quad \rho =| \mathbf{x}_e- \mathbf{x} |. \end{aligned}$$

Here, $\rho \in {\mathbb {R}}^3$ defines the distance from each point of the heart to a point placed at some distance from the ventricles (i. e., electrode location). This position was established at 2 cm away from the left ventricular wall, as is commonly defined to mimic pre-cordial leads to study T waves and QT intervals^65^. ECG signals were analyzed using Fourier Transform, from which power spectra were constructed, and the fundamental frequency was identified.

## Supplementary information

Supplementary Information.

## Abbreviations

HF: Heart failure
APD: Action potential duration
LV: Left ventricle
RV: Right ventricle
EPI: Epicardium
LVMM: Left ventricle mid-myocardium
NC: Normal heart
HFC: Heart with heart failure without biomaterial treatment
HFI: Heart with heart failure and biomaterial treatment
CL: Cycle lenght
AT: Sctivation time
RT: Reporalization time
GZ: Gray zone
IZ: Infarcted zone
NZ: Normal zone
GZR: Gray zone ratio
VF: Ventricular fibrillation
VT: Ventricular tachycardia
PDF: Probability density function
MRI: Magnetic resonance imaging
DT-MRI: Diffusion tensor magnetic resonance imaging.
