# Supplementary material for: In-silico study of the cardiac arrhythmogenic potential of biomaterial injection therapy
Source: Sci Rep. 2020 Jul 31;10:12990. doi: 10.1038/s41598-020-69900-4 (PMC7395773; doi:10.1038/s41598-020-69900-4)
Supplement: Supplementary file 1 — Supplementary Information. [file 41598_2020_69900_MOESM1_ESM.pdf]

In-silico study of the cardiac arrhythmogenic potential of biomaterial injection therapy

William A. Ramírez[1], Alessio Gizzi[2], Kevin L. Sack[3,4], Julius M. Guccione[3], and Daniel E. Hurtado[1,5,6*]

[1]Department of Structural and Geotechnical Engineering, School of Engineering, Pontificia Universidad Católica de Chile, Santiago, Chile.
[2]Nonlinear Physics and Mathematical Modeling Lab, Department of Engineering, Campus Bio-Medico University of Rome, Rome, Italy.
[3]Department of Surgery, University of California at San Francisco, San Francisco, CA, United States.
[4]Division of Biomedical Engineering, Department of Human Biology, University of Cape Town, Cape Town, South Africa.
[5]Institute for Biological and Medical Engineering, Schools of Engineering, Medicine and Biological Sciences, Pontificia Universidad Católica de Chile, Santiago, Chile.
[6]Millennium Nucleus for Cardiovascular Magnetic Resonance
[*]Corresponding author, e-mail: dhurtado@ing.puc.cl

SUPPLEMENTARY MATERIAL

	CL = 370 (ms)			CL = 504 (ms)			CL = 600 (ms)		
	NC	HFC	HFI	NC	HFC	HFI	NC	HFC	HFI
EPI	259	257	259	296	294	296	307	306	307
LV	264	269	264	304	312	304	316	325	316
RV	270	259	258	314	296	295	327	308	306
LVMM	288	291	291	338	342	343	354	358	359

Table 1. APD medians for different CL values taken as representative values of the restitution curve distribution, see Figure 1

	CL = 370 ms			CL = 504 ms			CL = 600 ms		
	NC	HFC	HFI	NC	HFC	HFI	NC	HFC	HFI
EPI	5.57	5.04	5.64	8.38	7.53	8.23	9.52	8.49	9.26
LV	6.87	9.58	9.23	9.71	13.51	13.08	10.85	15.16	14.68
RV	8.52	4.89	4.60	11.99	7.26	6.63	13.45	8.19	7.49
LVMM	5.99	7.47	7.23	8.63	11.56	10.82	9.74	13.41	12.35

Table 2. APD standard deviations for differencet CL values taken as representative values of the restitution curve distribution, see Figure 1

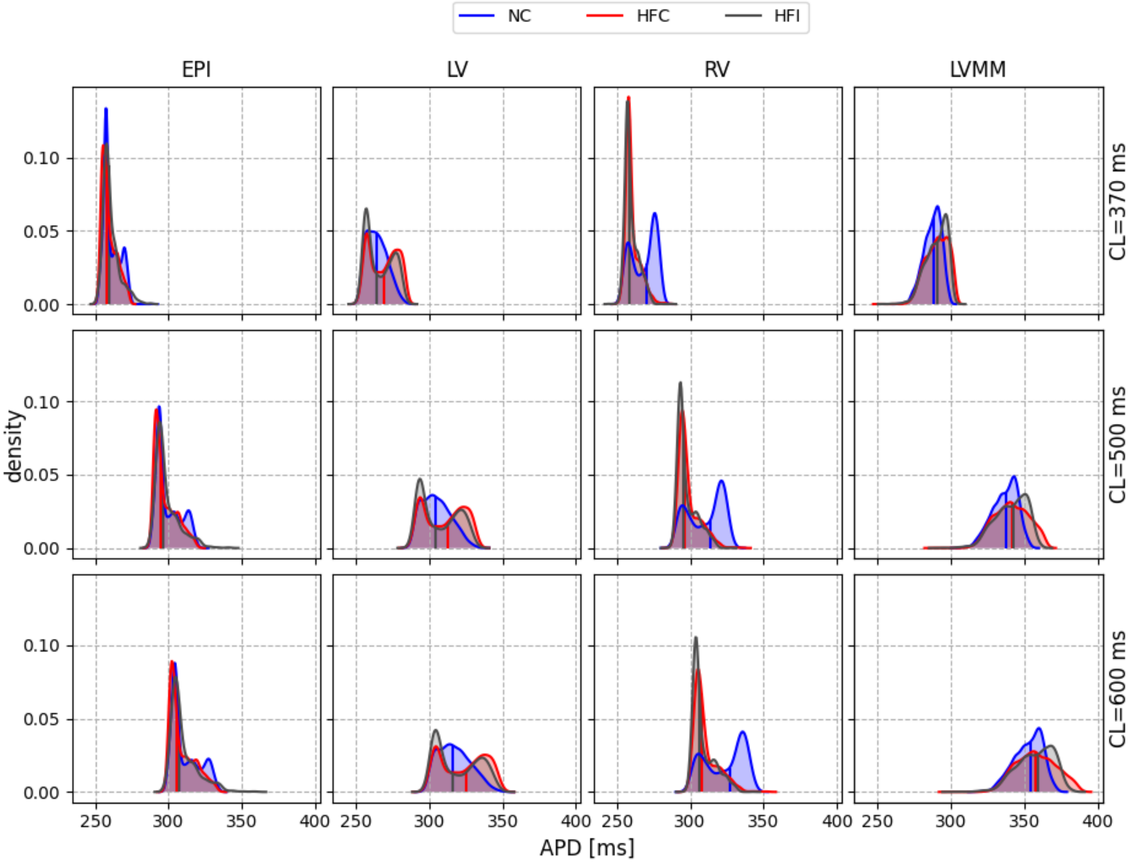

Figure 1. Density function plots of the APD restitution distribution for the three models at CL= 370 ms, CL= 500 ms and CL= 600 ms. This value are taken as representative values of the restitution curve distribution. The medians are plotted with vertical lines. Qualitative differences in the distribution may be seen at the RV and LV endocardium between the infarcted hearts and the normal control heart
